# The Role of Digital Opinion Leaders in Dengue Prevention Through Health Promotion and Public Health Collaboration: Qualitative Semistructured Interview Study

**DOI:** 10.2196/70997

**Published:** 2025-04-25

**Authors:** Andrew Green, Shishi Wu, Alberta Di Pasquale, Tikki Pang

**Affiliations:** 1 Regional Medical Affairs Vaccines, Growth and Emerging Markets, Takeda Pharmaceuticals International AG Singapore Branch Singapore Singapore; 2 IQVIA Real World Solutions Asia-Pacific Singapore Singapore; 3 Yong Loo Lin School of Medicine, National University of Singapore Singapore Singapore

**Keywords:** digital opinion leaders, dengue prevention, vaccine hesitancy, public health promotion, social media, health care providers

## Abstract

**Background:**

Dengue fever is a significant public health concern. The advent of social media has introduced digital opinion leaders (DOLs), health care professionals with substantial online followings who play a pivotal role in disseminating health information and combating misinformation.

**Objective:**

We aimed to investigate the role of DOLs in dengue prevention and explore their preferences for collaboration with health sector entities to strengthen dengue prevention initiatives.

**Methods:**

A qualitative study was conducted using semistructured interviews with 37 purposively selected DOLs from 8 countries in Latin America and Southeast Asia. They were selected based on their active online presence, dissemination of dengue-related content, and substantial social media followings. Interviews took place either in person or online, according to the participants’ chosen languages. Each session, lasting approximately 60 minutes, was audio recorded, transcribed verbatim, and subjected to thematic analysis to identify recurring themes.

**Results:**

The thematic analysis led to several key findings. First, DOLs used social media to enhance public health communication, focusing on raising awareness (16/37, 43%), correcting misconceptions (17/37, 46%), and modeling preventive behaviors (8/37, 22%) for infectious diseases. They educated audiences on disease symptoms and prevention, addressed vaccine hesitancy, and shared personal practices to encourage similar actions among followers. Second, 35% (13/37) of the DOLs reported a widespread lack of public knowledge about dengue and its prevention, with even less awareness of vaccine availability. In addition, 27% (10/37) of them identified challenges due to antivaccination sentiments and misinformation, while 8% (3/37) noted obstacles from perceived inadequate government leadership in dengue prevention. In response, DOLs leveraged their social media influence to educate the public. A significant number (22/37, 59%) of the DOLs emphasized the importance of regular promotion of vector control measures as the cornerstone of dengue prevention and 68% (25/37) highlighted the critical role of vaccines, particularly among vulnerable groups. Finally, collaboration was essential for expanding DOLs’ reach and credibility, with 54% (20/37) of them partnering with pharmaceutical companies, 43% (16/37) with government agencies, and 27% (10/37) with nongovernmental organizations. In these collaborations, 38% (14/37) of the DOLs emphasized the importance of adhering to ethical standards, and 57% (21/37) prioritized projects aligning with their personal values and professional standards, avoiding producing content that contradicted their beliefs or goals.

**Conclusions:**

DOLs are essential in disseminating dengue prevention information. They recognize their responsibility to raise awareness about dengue vaccines and dispel related misconceptions to combat vaccine hesitancy. Unlike nonmedical social media influencers, whose content may lack medical accuracy and be driven by monetization, DOLs provide evidence-based information, enhancing their credibility. Collaborations between DOLs and health sector stakeholders, although currently limited, hold significant potential for effective dengue prevention, provided they adhere to ethical standards and are supported by credible scientific evidence.

## Introduction

### Background

The emergence of social media and online platforms has given rise to a new group of influencers known as digital opinion leaders (DOLs). DOLs are influential figures who are able to shape the opinions and behaviors of their audience across a spectrum of fields through their activities on online platforms [1]. In the context of health care, key opinion leaders (KOLs) are traditionally recognized as experts within their professional fields, often gaining credibility through peer-reviewed publications, academic presentations, and advisory roles in health care and public health institutions. In recent years, a growing number of traditional KOLs have been transitioning into DOLs through their increasing digital presence, while some DOLs, such as early-career professionals, primarily establish their influence online rather than in academia or professional settings [1]. Similar to KOLs, DOLs are usually qualified health care providers [2]. However, in contrast to traditional KOLs, who primarily communicate and influence via conventional channels, such as journal publications or speaking activities, DOLs operate regularly within the digital domain. This enables them to exert influence over a wider and more diverse audience, spanning various communities, including both health care providers and the general population [1,3]. While both DOLs and social media influencers (SMIs) operate on online platforms and engage with diverse audiences, DOLs are a distinct subset of SMIs, characterized by their medical qualifications and commitment to educating the public with evidence-based health information. In contrast, SMIs often lack formal medical training and primarily focus on lifestyle content, trends, and personal experiences, which may not always be grounded in scientific evidence [4]. Table 1 provides an overview of the comparison between KOLs, DOLs, and SMIs.

**Table 1:** Comparison of key opinion leaders (KOLs), digital opinion leaders (DOLs), and social media influencers (SMIs). This table compares the roles, expertise, content focus, audience engagement, motivations, and health sector collaborations of KOLs, DOLs, and SMIs in health communication.

Characteristic	KOLs	DOLs	SMIs
Definition	Established experts in their field, typically with a strong academic, clinical, or research background	Health care professionals with a strong digital presence who use social media to educate and engage audiences on health topics	Individuals with a large online following who influence public opinions and behaviors, often lacking formal medical expertise
Influence medium	Academic journals, conferences, advisory boards, and professional networks	Social media platforms (eg, YouTube, Instagram, TikTok, LinkedIn, Facebook, and X [formerly known as Twitter])	Social media platforms (eg, YouTube, Instagram, TikTok, LinkedIn, Facebook, and X)
Content focus	Evidence-based discussions, research findings, and policy recommendations	Evidence-based discussions, research findings, policy recommendations, health education, lifestyle, and personal experiences	Lifestyle, trends, and personal experiences, often with commercial sponsorships
Engagement with followers	Limited direct interaction; influence occurs through professional channels	High engagement, with active discussions; Q&A^a^ sessions; and content tailored to their audience	High engagement, with active discussions; Q&A sessions; and content tailored to their audience

^a^Q&A: question and answer.

Recent evidence suggests that leveraging digital platforms holds promise for promoting a range of health behaviors to achieve positive health outcomes [5-7]. For example, SMIs championed and successfully promoted the adoption of preventive measures, including the use of masks, testing, and vaccination during the COVID-19 pandemic in many countries, such as the United States and Finland [8-10]. Despite the many studies on SMIs, those specifically exploring the role of DOLs are limited. One review that examined the use of online influencers to improve the uptake of human papillomavirus vaccines suggested that those with a health or medical background can serve as credible information sources and engage their audience in positive conversations about human papillomavirus, thereby fostering positive attitudes toward human papillomavirus vaccines and countering misinformation [11]. However, the study did not distinguish the definitions of DOLs and SMIs or further highlight their differences [11].

Furthermore, SMIs may offer an avenue for preventing complex diseases, such as dengue. Dengue stands as a major public health challenge, with a substantial global disease burden. Dengue incidence has increased ten-fold in the last 2 decades [12]. According to an update from the World Health Organization (WHO) in 2024, dengue is endemic in >100 countries, putting approximately 3.9 billion individuals at risk of infection [12,13]. Latin America and Southeast Asia are among the regions most affected by dengue globally [14,15]. The average dengue-related cost was reported to be US $2.1 billion annually in the Americas, surpassing the financial burden of many other viral diseases [16]. Particularly, a dengue outbreak in Brazil resulted in more than half a million recorded cases within the first 2 months of 2024 [17]. In Southeast Asia, a study estimated that 2.9 million dengue cases and >5000 deaths occurred each year in the 2000s, with an annual economic burden of US $950 million [18].

Given the complex dynamics of dengue transmission, which involve vectors, humans, and the environment [19,20], effective dengue prevention requires a combination of vaccines and vector control measures [21]. Due to the lack of widely available dengue vaccines, vector control remains the primary strategy for dengue prevention. However, community-based approaches to vector control rely heavily on the knowledge and behaviors of individuals within the community [22]. Likewise, upon the availability of a dengue vaccine, its uptake would also hinge upon the public’s awareness and understanding to facilitate its swift adoption among populations.

Over the past 75 years, dengue vaccine development has proven difficult, with only live attenuated virus vaccines achieving licensure or phase 3 status [23]. The first dengue vaccine (CYD-TDV) was licensed in 2015, but after its launch, updated data showed an increased risk of severe outcomes in vaccine recipients without a previous dengue infection compared to their unvaccinated counterparts [24]. The WHO soon revised its recommendation that CYD-TDV should only be used in previously infected individuals [25]. In the Philippines, where this vaccine was introduced into the national immunization program, there was a controversy regarding this vaccine’s safety [26]. This controversy led to increased hesitancy toward not only dengue vaccines but also COVID-19 vaccines and routine immunization in the country [25-27]. Recently, the approval of a 2-dose vaccine named TAK-003 in several countries has marked a significant advancement in dengue disease prevention, where it could be used regardless of previous exposure [28,29]. In 2024, the WHO fulfilled its advisory role by issuing a position paper on dengue vaccines tailored for large-scale vaccination initiatives [30]. Within this directive, the WHO recommends the administration of TAK-003, particularly targeting children aged 6 to 16 years, especially in regions with high dengue transmission rates [30,31]. This recommendation gains further importance against the backdrop of escalating dengue cases reported in endemic areas, underscoring the urgent need for additional preventive measures.

Consequently, evidence-based guidance is needed to inform the development of vaccination education strategies involving DOLs, particularly given the scientific complexity of dengue and past controversies surrounding dengue vaccines. While SMIs have been involved in infectious disease prevention previously, the specific role of medically qualified DOLs in dengue prevention and how they differ from SMIs lacking medical backgrounds remains unexplored.

### Objectives

This study aims to examine the role and practice of DOLs in influencing dengue awareness and prevention on social media platforms. In addition, we investigated DOLs’ preferred modes of collaboration with other stakeholders in the health sector on dengue prevention.

## Methods

### Study Design and Participant Recruitment

We used a qualitative study approach to address the study objectives. Specifically, we conducted semistructured interviews with purposively selected DOLs in 8 countries in Latin America (Argentina, Brazil, and Colombia) and Southeast Asia (Indonesia, Malaysia, the Philippines, Singapore, and Thailand), where dengue is endemic.

We purposively recruited DOLs who were qualified health care providers and have been actively disseminating information on infectious disease prevention (including dengue) and vaccination online. Specific inclusion criteria are listed in Textbox 1. Web-based research was conducted by the research team to identify eligible DOLs in each country. On the basis of the inclusion and exclusion criteria, we selected and compiled a list of potential study participants who met the inclusion criteria in each country. The research team contacted potential participants either using their contact information available online or via their social media accounts to obtain initial consent to participate in our study.

**Inclusion criteria for digital opinion leaders eligible for interviews in a semistructured qualitative study on dengue prevention efforts in Latin America and Southeast Asia. Study participants were selected and recruited based on these criteria between March and August 2024 across 8 dengue-endemic countries: Argentina, Brazil, Colombia, Indonesia, Malaysia, the Philippines, Singapore, and Thailand.**

**Inclusion criteria for individuals**

- Qualified physicians who were authorized to provide health services due to their qualifications, certifications, and practical experience, as well as those who had a medical degree but were not currently practicing, and health scientists and educators with a medical degree or professional medical credentials
- Those who had been active online in the past 6 months on at least 2 of the following social media platforms: YouTube, LinkedIn, TikTok, Instagram, X (formerly known as Twitter), Facebook, or other Blogs
- Those who had shared content on prevention and vaccination related to dengue
- Those who had >10,000 followers across all social media platforms

### Data Collection

In each country, interviews were conducted by female facilitators who had bachelor’s degrees in communication or health-related majors and extensive experience in qualitative research. To minimize interviewer bias and ensure consistency in interview delivery, all interviews were moderated by the same facilitator in each country, and the DOLs did not have established relationships or previous knowledge of the interviewers. A topic guide was used to facilitate the interviews (Multimedia Appendix 1). In Singapore, Malaysia, and the Philippines, interviews were conducted in English. In Indonesia, Thailand, Brazil, Argentina, and Colombia, interviews were conducted either in English or the local languages, depending on participants’ preferences. The topic guide was translated into local languages and reviewed by the research team before the interviews to ensure translation accuracy. A briefing session was conducted with all the facilitators to ensure they were thoroughly familiar with the objectives and methods of the study as well as the interview process and the questions in the topic guide.

The DOLs were initially contacted via email or direct messages through their social media platforms to seek their consent for participation in this study. Interviews were conducted either online (using video or audio calls) or in person, based on the participants’ preferences. Each interview lasted approximately 60 minutes and was audio recorded, with field notes taken concurrently. The interviews commenced with participant introductions and proceeded to cover three main areas: (1) the DOLs’ journey and engagement strategies with their followers; (2) their experiences and current practices in disseminating health information, particularly on dengue and other infectious disease prevention; and (3) the strategies and approaches they used or preferred for collaborating with various stakeholders in the health sector. In this study, the health sector is defined to encompass a broad range of stakeholders involved in or influencing the delivery of health care services or products, such as health care providers, public health agencies, the pharmaceutical industry, nongovernmental organizations (NGOs), and government regulatory agencies [32,33]. All audio-recorded interviews were transcribed verbatim. Transcriptions of interviews conducted in languages other than English were translated into English. None of the recruited DOLs withdrew from the study. Transcripts were not returned to the DOLs for their review or correction.

### Data Analysis

We used a thematic analysis approach to analyze the data generated from the interviews [34]. Adopting an interpretivist lens, we focused on participants’ experiences, perceptions, and understanding of the discussed topics [35]. After data familiarization, 2 researchers coded the first 5 transcripts inductively and independently. They compared the codes regularly to ensure intercoder reliability and identified emerging themes. Disagreement was resolved through discussion and consultation with a third researcher who could provide neutral views on the analyzed data. Once a coding frame was developed and agreed upon, it was applied to the remaining transcripts to ensure consistency. The codes were refined and grouped into subthemes and main themes iteratively. The coding frame was finalized after analyzing all transcripts. The 2 researchers reviewed the themes to ensure alignment with the research questions and refined them as necessary. The analysis was conducted using Microsoft Excel. The findings were not returned to the DOLs for their review or feedback. The study methods and results were reported following the COREQ (Consolidated Criteria for Reporting Qualitative Research) checklist (Multimedia Appendix 2) [36].

### Ethical Considerations

This study received an exemption from the Pearl Institutional Review Board (reference number 2023-0424). Informed consent was obtained through a participant’s dated signature. For web-based interviews, the consent form was emailed to the participant before the interview. Participants provided their electronic signatures and returned the form via email. Data collected from the interviews were deidentified to ensure confidentiality, and access to these digital files was restricted to members of the research team. Participants received an honorarium based on the fair market value in each country as governed by the country’s pharmaceutical regulation and fair market value.

## Results

### Overview

A total of 94 DOLs were identified to be eligible and approached, of which 37 (39%) gave their consent to participate in this study. As shown in Table 2, among these 37 DOLs interviewed, 24 (65%) were from Southeast Asia and 13 (35%) from Latin America, with a nearly equal distribution of female (n=17, 46%) and male (n=20, 54%) participants. Most DOLs were pediatricians (n=17, 46%) and had <20 years of professional experience (n=19, 51%). Most participants had been active online as DOLs for 3 to 5 years (n=16, 43%) or 5 to 10 years (n=11, 30%). The most commonly used social media platforms included TikTok (n=32, 86%), Instagram (n=29, 78%), Facebook (n=19, 51%), and YouTube (n=16, 43%). Across all platforms, most (27/37, 73%) participants had follower counts ranging from 14,000 to 300,000, with 6 (16%) participants having >600,000 followers.

**Table 2:** Demographic characteristics of digital opinion leaders (DOLs) interviewed in a semistructured qualitative study on dengue prevention efforts in Latin America and Southeast Asia (N=37).

Characteristics				Study participants, n (%)
**Sex**				
	Female		17 (46)	
	Male		20 (54)	
**Specialty**				
	Pediatrician		17 (46)	
	General practitioner		7 (19)	
	Infectious disease specialist		4 (11)	
	Internal medicine specialist		4 (11)	
	Gynecologist		1 (3)	
	Public health medicine specialist		1 (3)	
	Emergency medicine specialist		1 (3)	
	Pulmonologist		1 (3)	
	Medical technologist		1 (3)	
**Experience (y)**				
	<10		10 (27)	
	10-20		9 (24)	
	20-30		3 (8)	
	>30		2 (5)	
	Not mentioned		13 (35)	
**Region**				
	**Southeast Asia**		24 (65)	
		Indonesia	6 (16)	
		Malaysia	5 (14)	
		Singapore	1 (3)	
		Thailand	6 (16)	
		The Philippines	6 (16)	
	**Latin America**		13 (35)	
		Argentina	3 (8)	
		Brazil	6 (16)	
		Colombia	4 (11)	
**Types of platforms**				
	TikTok		32 (86)	
	Instagram		29 (78)	
	Facebook		19 (51)	
	YouTube		16 (43)	
	LinkedIn		3 (8)	
	X (formerly known as Twitter)		2 (5)	
	Blogspot		1 (3)	
	Other (website and podcast)		2 (5)	
**Followers, n**				
	14,000-50,000		9 (24)	
	50,001-100,000		7 (19)	
	100,001-300,000		11 (30)	
	300,001-600,000		3 (8)	
	600,001-900,000		2 (5)	
	>900,000		4 (11)	
**Duration of being active as a DOL (y)**				
	<3		6 (16)	
	3-5		16 (43)	
	5-10		11 (30)	
	Not mentioned		4 (11)	

DOLs primarily targeted a diverse audience through their content creation efforts, aiming to educate and engage individuals across various demographics. Of the 37 DOLs, many reported that their primary audience was the general public (n=23, 62%) and predominantly women (n=20, 54%). Most (n=32, 86%) DOLs mentioned that their followers were aged between 18 and 40 years. Some (n=10, 27%) DOLs, particularly those from Latin American countries, also indicated that they were followed by health care providers. As a result, the content created by the DOLs was often tailored to resonate with both laypersons and health care professionals, seeking to bridge gaps in public understanding while also facilitating knowledge exchange among health professionals.

As shown in Multimedia Appendix 3, the five emerging themes were organized into three sections to address the study objectives: (1) the increasing influence of DOLs in infectious disease prevention, (2) current practices of DOLs contributing to dengue prevention, and (3) exploring ways of collaboration to strengthen dengue prevention. The corresponding quotes supporting each theme are also presented in Multimedia Appendix 3.

### Section 1: The Increasing Influence of DOLs on Infectious Disease Prevention (Influence of DOLs on Infectious Disease Prevention)

The DOLs participating in this study acknowledged that the rise of social media and the increasing presence of health care providers in the digital space had changed how individuals sought and accessed health information. Currently, the DOLs contribute to the prevention of infectious diseases on online platforms through 3 primary pathways: raising public awareness, dispelling misconceptions, and serving as role models.

Many (16/37, 43%) DOLs perceived their primary role as raising public awareness about infectious diseases and their prevention. Leveraging the power of social media, DOLs aimed to educate the public on crucial health topics, including the recognition of common signs and symptoms of diseases and the appropriate timing for seeking medical care. Recognizing that building awareness was a gradual process, DOLs often began with simple, relatable content to encourage informed behaviors. For instance, they used notable cases, including those of prominent individuals with diseases such as dengue, to underscore the severity of certain conditions and advocate for preventive measures, such as vaccination. However, DOLs acknowledged that while online information dissemination could enhance knowledge, translating this knowledge into actionable behaviors was not guaranteed.

Many (17/37, 46%) DOLs mentioned that they actively addressed misconceptions surrounding infectious disease prevention, especially regarding vaccines. They tackled community concerns, such as fears of vaccines causing autism or other adverse events, by producing content that provided explanations and clarifications on these issues. Through their efforts, they aimed to educate the public and alleviate concerns. In addition, these DOLs emphasized that while vaccines are crucial for disease prevention, they are just one aspect of a comprehensive strategy that should also include everyday preventive activities. Furthermore, they addressed misunderstandings regarding vaccine efficacy and corrected the misconception that vaccination provided absolute immunity, as illustrated in the following quote:

For example, some people understand that once you are vaccinated you will not get sick, but this is incorrect. Actually, you can still get sick, but your symptoms would not be too severe. I want to get the fact out.

ID17, Southeast Asia

Furthermore, some (8/37, 22%) DOLs mentioned serving as role models for infectious disease prevention. Through their own health-promoting behaviors, they influenced their audiences to adopt similar practices. For example, DOLs shared personal practices, such as placing sand in plant dishes to prevent mosquito breeding, thereby educating and motivating their followers to take similar preventive actions. In addition, some (3/37, 8%) DOLs shared their experiences with receiving influenza or COVID-19 vaccinations to encourage vaccination uptake among their audience.

### Section 2: Current Practices of DOLs Contributing to Dengue Prevention

#### Theme: Current Challenges in Dengue Prevention and Control

All (37/37, 100%) DOLs acknowledged the importance of addressing dengue and identified three primary challenges in dengue prevention: (1) inadequate public awareness and knowledge of dengue and its preventive measures; (2) insufficient governmental initiatives; and (3) actions to combat dengue, antivaccination sentiments, controversies, and the spread of false information.

Some (n=13, 35%) DOLs observed a widespread lack of knowledge and awareness about dengue and its prevention measures among the public despite extensive public awareness campaigns in many countries. They opined that while the campaigns have equipped populations with a fundamental understanding of dengue, especially in endemic regions, they have not effectively conveyed the potential for severe sequelae associated with the disease. Consequently, the preventive measures practiced by the public may not be optimal. Some (9/37, 24%) also suggested that this could be attributed to public disinterest or fatigue toward messages about traditional dengue prevention measures, such as vector control, which had been promoted for decades. This point can be demonstrated by the following quote:

Traditional vector prevention measures, such as avoiding stagnant water and everything, are already well-known. I think that people have grown tired of this message, especially since we’ve been discussing this for 40 years without making any progress.

ID8, Latin America

In addition, few (3/37, 8%) DOLs noted potential challenges in dengue prevention related to the level of government involvement. While acknowledging the importance of government leadership in prevention efforts, they expressed hope for greater collaboration and proactive measures from governmental authorities to address the issue effectively.

Even less awareness exists among the public regarding the availability of a vaccine for dengue. Compounding this issue, many (10/37, 27%) DOLs perceived significant challenges in dengue prevention due to antivaccination sentiments, controversies, and the spread of fake news. They observed that many individuals were reluctant to engage with information about vaccines and that the rise of antivaccine movements posed obstacles to introducing dengue vaccines. Furthermore, some (8/37, 22%) DOLs noted challenges when discussing adverse effects associated with dengue vaccines, particularly due to public skepticism and fear stemming from past controversies over vaccines:

Our difficulty now is to get people to reduce the anti-vaccine movement because we come from a few years in which this gets much worse. It will be a battle to reinforce the population to trust vaccines again.

ID5, Latin America

#### Theme: Topics to Discuss for Dengue Prevention and Sources of Information

Noting these challenges, DOLs recognized the value of leveraging their influence on social media for educational purposes, as they had been promoting vector control strategies and raising awareness of dengue symptoms online. Most (22/37, 59%) DOLs agreed that vector control remained the cornerstone of dengue prevention and should be promoted regularly. DOLs strongly emphasized the need to educate people about vector control measures, such as eliminating breeding sites and using mosquito repellents, especially in known dengue hot spots. However, the challenge lay in how to effectively promote these measures to capture public interest, especially among those who were fatigued by repetitive vector control messages:

We need to have better campaigns for the population to talk about how to control the vector, which is very difficult.

ID6, Latin America

In addition to vector control, most (25/37, 68%) DOLs unanimously underscored the pivotal role of vaccines in mitigating the burden of dengue, especially among vulnerable populations. The DOLs in both regions identified vaccine hesitancy as a significant barrier to rolling out dengue vaccines, considering the past controversies and conflicting information available online, which often resulted in public uncertainty and skepticism. As a result, many (14/37, 38%) DOLs acknowledged the importance of enhancing awareness regarding dengue vaccines and providing online education on their efficacy and safety. Some (2/37, 5%) recognized their responsibility in dispelling misconceptions through online engagement with their audience to counter vaccine hesitancy:

We can create content debunking myths on vaccines…We need to change their mindsets, so we explain why their belief is wrong.

ID32, Southeast Asia

Finally, many (13/37, 35%) DOLs mentioned the importance of disseminating information about the application of new technologies in dengue prevention. For example, DOLs from Malaysia highlighted the integration of dengue tracking into the MySejahtera app in Malaysia, originally developed for tracking COVID-19 clusters, as a valuable innovation for real-time monitoring of dengue cases. The implementation of Wolbachia, trialed in Indonesia, Malaysia, and Singapore to diminish mosquitoes’ ability to transmit dengue, was also referenced.

Furthermore, several (4/37, 11%) DOLs pointed out the importance of timing when posting dengue-related content. They tended to share information about dengue during rainy seasons or outbreaks when dengue cases were typically more prevalent because it aligned with the public’s increased interest in dengue:

The content that you want to present must be as interesting as possible. Also, the timing should be right. The timing is right now during the rainy season, so it’s appropriate [to share dengue-related content].

ID15, Southeast Asia

When queried about the sources of information frequently used to generate content, most (27/37, 73%) DOLs emphasized their heavy reliance on scientific journals and authoritative websites. Journals were esteemed for their credibility, adherence to international standards, and verified content, rendering them the primary source of information. Resources such as PubMed, UpToDate, Medscape, and WebMD were commonly accessed for the latest medical information. In addition, many (14/37, 38%) DOLs cited textbooks and guidelines published by national or international authorities, such as the ministry of health or WHO, as valuable sources.

### Section 3: Exploring Ways of Collaboration to Strengthen Dengue Prevention

#### Theme: Types of Collaboration

The DOLs expressed a willingness to collaborate with diverse stakeholders within the health sector, as they cited examples of past engagements with various entities covering a range of health topics. They had primarily partnered with pharmaceutical companies for health education and raising awareness about products (20/37, 54%). Many (10/37, 27%) DOLs had collaborated with NGOs to promote nutrition and hygiene practices. Many (16/37, 43%) had collaborated with government and health agencies on public health education initiatives, which included dengue. However, only a few (2/37, 5%) DOLs were involved in collaborations to promote dengue prevention specifically. In terms of their roles in collaboration, some (6/37, 16%) DOLs indicated that they preferred and, in many cases, were tasked with creating technically oriented content. In their past collaboration with government agencies, the DOLs (3/37, 8%) created posts or videos, which would then undergo review by the government before being shared online. This ensured alignment with collaboration objectives and organizational requirements.

#### Theme: Factors Considered for Collaboration

Factors commonly reported by DOLs to influence their decision on collaboration included adherence to ethical standards, alignment with their values, and the credibility of scientific evidence supporting the initiatives. A few DOLs also discussed their preferences regarding compensation in the collaboration.

Many (14/37, 38%) DOLs emphasized the importance of adhering to ethical standards when considering collaborations. They were committed to avoiding topics that touched on sensitive issues, such as ethnicity, religion, or race, focusing instead on content that was universally beneficial and respectful. The DOLs (16/37, 43%) exercised caution regarding the direct promotion of health products when collaborating with pharmaceutical companies and were mindful of restrictions imposed by professional ethical boards, committing to operate within these boundaries to uphold their credibility and professional integrity:

If the campaign is focused on educating people about the disease rather than selling the product, [it’s fine]. If it’s too product-centred, I would say no. We make sure the campaign is all about education.

ID29, Southeast Asia

Most (21/37, 57%) DOLs prioritized projects that aligned with their personal values and professional standards. They were unwilling to compromise on content that contradicted their beliefs or goals, such as vaccine endorsements that did not align with their scientific understanding:

[It depends on] if we have the same goal or not. Also, if my role would be benefiting for the course or not, if not, then I will not work with them.

ID18, Southeast Asia

Moreover, many (19/37, 51%) DOLs acknowledged the importance of credible scientific evidence in their decision-making processes. They mentioned that when collaborating with pharmaceutical companies, they requested comprehensive information on pharmaceutical products, including adverse effects and scientific evidence, before agreeing to the collaboration. Such measures helped to ensure the accuracy of information disseminated by DOLs.

Finally, our observations consistently revealed a preference among most (27/37, 73%) DOLs for financial compensation to reflect their time and efforts, alongside an expectation of due acknowledgment. They disclosed receiving monetary payments from collaborators, notably pharmaceutical companies, or expressed a distinct inclination toward financial remuneration via honoraria for speaking engagements or direct payment for content creation and collaborations. However, it is worth noting that this study did not delve into the specifics of honoraria rates.

## Discussion

### Principal Findings

In this qualitative study, we found that online influencers, including qualified health care professionals (DOLs), have become an important avenue for public health education, particularly concerning the prevention of infectious diseases, such as dengue. Although traditional vector control methods remain essential and are actively endorsed by DOLs, the public’s attention can wane due to message fatigue. Both the public and DOLs are interested in new methods for dengue prevention, such as vaccines and new vector control technologies, such as Wolbachia. DOLs recognize the need to increase awareness and deliver education about dengue vaccines. They are aware of their role in debunking misconceptions and controversies about dengue vaccines in the digital space as a means to tackle vaccine hesitancy.

Our findings align with several well-established communication theories, offering a conceptual foundation for understanding their role in health promotion and communication. First, our findings align with the diffusion of innovations theory, which describes how new ideas and health information spread through communication channels before reaching the public [37]. In the case of DOLs, they act as intermediaries who disseminate evidence-based messages to their online audiences. Second, our findings align with the two-step flow theory, which suggests that media messages are not directly absorbed by the public but instead filtered through opinion leaders who reinterpret and disseminate information [38]. Previous research examining the role of SMIs in public health communication during the COVID-19 pandemic found that SMIs served as information mediators, translating and personalizing public health messages for their audiences while shaping social norms around pandemic behaviors [9]. This study reinforces the model by demonstrating how DOLs, akin to SMIs, adapted official health messages to enhance accessibility and engagement [9]. By effectively translating complex health information, DOLs facilitated the adoption of preventive behaviors, such as dengue vaccination and vector control measures, among their online audiences.

While DOLs and SMIs share overlapping roles in health communication, there are notable distinctions that can impact the weight and trustworthiness of their messages. First, misinterpretations by SMIs can arise due to their lack of formal medical training, as medical information can be complex and confusing without proper expertise [39,40]. Instead of delivering content based on rigorous evidence, SMIs often focus on fostering engagement with their audience that is more emotion driven with less emphasis on factual accuracy. This can sometimes lead to the inadvertent spread of misinformation, contributing to antivaccination movements and vaccine hesitancy, particularly during the COVID-19 pandemic [41-43]. This highlights the importance of medical training in accurately interpreting and conveying health information. DOLs, as medically trained professionals, use information from credible and authoritative sources to create their content, ensuring the validation of information and guiding their audience toward reliable sources [44]. There is substantial evidence supporting the positive impact of SMIs on health promotion, but the additional medical expertise of DOLs enhances the reliability of the health information they share.

Second, the motivations driving content creation can differ among influencers. Many SMIs leverage their influence for monetization through advertisements and brand endorsements, a common practice in the marketing and advertising industries, which can sometimes influence the type of content they produce [45-47]. In contrast, DOLs, who are also part of the broader SMI community, are typically bound by strict medical and ethical codes to adhere to unbiased, evidence-based practices in their posts. Our research supports this, showing that DOLs often avoid direct product endorsements, instead focusing on promoting scientifically backed health knowledge and behaviors. This approach helps build trust with their audience and enhances their credibility as sources of online health information.

Third, the ability of DOLs to navigate scientific and medical literature makes them valuable partners in health promotion activities. This is particularly true for complex diseases, such as dengue, where both the public and DOLs show a keen interest in novel approaches, such as vaccination and new vector control technologies. These approaches underscore the necessity for reliable and up-to-date information, which often can only be accessed through collaborations with ministries, recommending bodies, and vaccine manufacturers. Although collaborations within the health sector for dengue prevention remain limited, our study demonstrated that DOLs are receptive to engaging with diverse entities, provided these partnerships adhere to ethical standards and are backed by credible scientific evidence. These collaborations enable DOLs to align on best practices and public health priorities while staying informed about the latest technologies and developments in dengue prevention and treatment.

Expanding on the aforementioned information, we also noted differences in collaborative approaches between SMIs and DOLs. For instance, when government agencies engaged SMIs in campaigns for promoting influenza vaccines or raising awareness about COVID-19, SMIs were given fact-checked information to share directly or incorporate into their content [9,48]. Although this approach ensures the accuracy of shared information, studies argue that it does not fully leverage an influencer’s unique content creation abilities or reflect their persona, potentially undermining the impact of SMIs in health campaigns [49]. In addition, SMIs were sometimes not required to respond to comments by campaign sponsors, as the sponsors provided sample responses or guidelines for SMIs to engage with their audience and respond to technical questions [48,50]. In contrast, medically trained DOLs often enjoy more creative freedom. On the basis of our study, DOLs are frequently tasked with creating technical-oriented content. Furthermore, they are encouraged to actively counter misinformation on online platforms, engage in online forums to address queries, and monitor online discussions and sentiments about vaccines, thereby contributing to shaping a well-informed public perception of dengue vaccines [51,52].

More importantly, DOLs’ online activities can complement offline dengue prevention efforts. They can participate in dengue awareness and education campaigns led by NGOs or government health authorities, thereby improving reach and engagement among young, active online users. This can augment traditional dengue prevention campaigns, which often face challenges of limited reach and sustainability due to resource constraints for physical campaign activities, particularly in low- and middle-income countries [53].

Furthermore, as social media emerges as a primary source of health information, including vaccine-related content [54-56], the role of DOLs becomes increasingly crucial in addressing vaccine hesitancy by combating online misinformation and disinformation [51,57]. This is particularly relevant for raising awareness about dengue vaccines and the potential introduction of new dengue vaccines in the near future. DOLs can be leveraged to support vaccine advocacy efforts and tackle vaccine hesitancy through collaboration with various stakeholders, including national or regional health authorities, international organizations, pharmaceutical companies, and NGOs.

### Recommendations for Best Practices in Leveraging DOLs in Dengue Prevention

**Figure 1:**
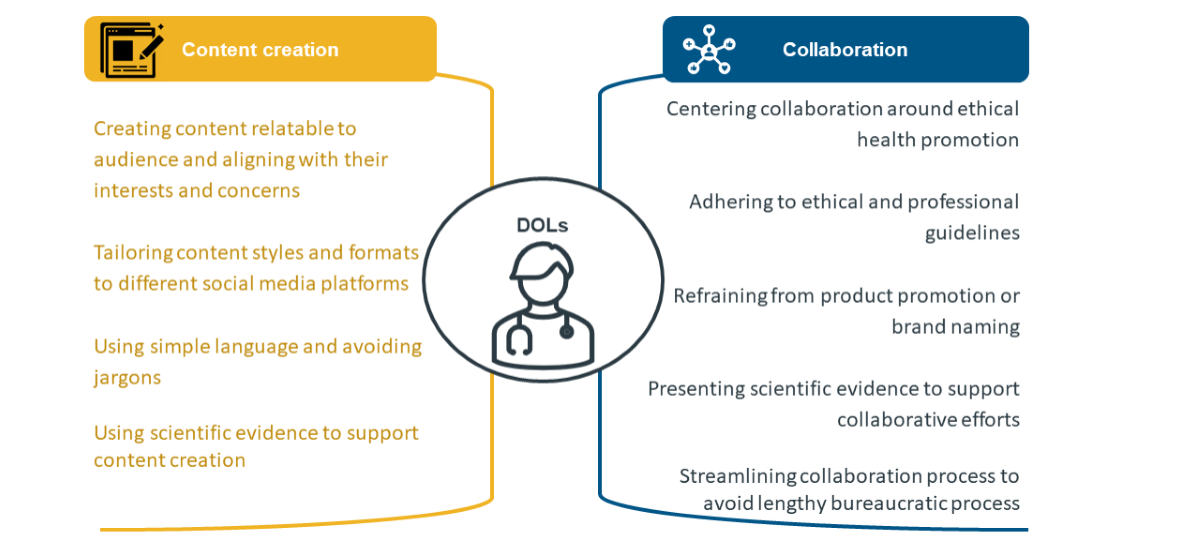
Recommendations for best practices in leveraging digital opinion leaders (DOLs).

Leveraging their online influence and credibility, DOLs are invaluable in raising awareness about dengue preventive measures. They can play a crucial role in vaccine educational efforts by disseminating accurate information about vaccines and addressing hesitancy, especially with the introduction of a new dengue vaccine. To optimize the effectiveness of DOLs in dengue prevention strategies, we propose the following recommendations for best practices focusing on 2 key areas: content creation and collaboration (Figure 1).

First, to enhance the effectiveness of health communication, it is crucial to identify key aspects essential for understanding dengue and its prevention. Dengue is a complex disease with a wide spectrum of clinical presentations and intricate transmission dynamics involving vectors, humans, and the environment [19,20,58]. In addition, the nuanced considerations in the use of dengue vaccines, including differing recommendations based on previous exposure status and the anticipated effects of the vaccines, necessitate clear and accessible communication to prevent confusion and address vaccine hesitancy among the public [59-61]. To optimize the effectiveness of content created by DOLs, it is essential to tailor it to specific social media platforms and ensure that it resonates with the audience. Simplifying complex medical concepts and scientific evidence without resorting to jargon is vital for ensuring accessibility to a wide audience. Furthermore, citing references and sources can bolster the credibility of the content and educate the audience about reliable health information resources.

Second, collaborations between DOLs and other health sector stakeholders should focus on the ethical promotion of health behaviors or education rather than endorsing specific brands or products. This is particularly relevant when DOLs engage with pharmaceutical companies. Obtaining information about novel vaccines, such as those for dengue, can be challenging, and accessing such information may require direct engagement with pharmaceutical companies. To facilitate DOLs in upholding ethical and professional standards, collaborations between DOLs and pharmaceutical companies could involve training sessions to ensure accurate and informed posting of vaccine-related information. It is important to present scientific evidence that supports partnership efforts, emphasizing an evidence-based approach. In addition, given that dengue prevention is often a government-initiated priority, partnering with government agencies for public education initiatives and aligning with their strategies can be beneficial for effectively disseminating information about dengue prevention and vaccination. DOLs should consciously avoid endorsing or naming specific products in their content but focus on promoting prevention measures and raising awareness about dengue. However, such collaboration with government authorities is sometimes challenged by the lengthy process of obtaining approvals. Streamlining the approval process would make these collaborations more efficient and help avoid unnecessary delays.

### Strengths and Limitations

To our knowledge, this is the first qualitative study to explore the role and practices of DOLs with medical credentials in the domain of dengue prevention. Our findings support the engagement of DOLs as an innovative channel in public health promotion that could significantly enhance dengue prevention initiatives. Moreover, the recruitment of DOLs from dengue-endemic regions ensures the representativeness of our study participants in discussing dengue prevention strategies. The inclusion of participants from a wide range of countries helped enrich the data with opinions from different perspectives. Finally, data saturation was achieved, with no new codes or themes emerging after the analysis of 12 transcripts.

Our study has several limitations. First, we focused exclusively on DOLs specializing in communicable diseases and vaccination, which may limit the generalizability of our findings to other health topics. Future research exploring DOLs’ roles across different disease contexts could provide a broader understanding of their impact. In addition, selection bias may be present, as we recruited DOLs with substantial social media followings due to their wider reach and potential for public health collaboration. While this approach captured insights from those with the greatest audience influence, it may have excluded perspectives from DOLs with smaller platforms, who might use different engagement strategies. Second, as with all qualitative research, our findings are subject to interpretation. To ensure rigor, we used a structured topic guide, conducted interviews using experienced qualitative researchers in each country, held facilitator briefing sessions to align methodological approaches, and engaged multiple researchers in data analysis to reduce bias [62]. Third, while thematic saturation was not a predetermined sampling criterion, we observed inductive data saturation, as no new themes emerged after analyzing 12 interviews [63]. Fourth, member checking was not conducted due to practical constraints. Re-engaging health care professionals across 8 countries would have imposed a significant logistical burden, likely leading to low response rates and selection bias. In addition, multilingual translations could have introduced inconsistencies, while the additional time and financial resources required for postanalysis engagement made member checking unfeasible within the study’s scope. Finally, our findings indicate limited engagement of DOLs in dengue prevention collaborations with other health sector stakeholders. This may reflect insufficient governmental initiatives for dengue prevention and control, as reported by the DOLs in our study and previous research [64,65]. Future studies incorporating perspectives from government agencies responsible for communicable disease prevention are needed to address this knowledge gap.

### Conclusions

As a credible health information source, DOLs can be instrumental in raising awareness about dengue preventive measures and introducing new dengue prevention strategies, including dengue vaccines, particularly to the digitally engaged population that often seeks health information online. Recognizing the multifaceted nature of dengue prevention, collaborative endeavors involving DOLs should prioritize the ethical promotion of health knowledge and behaviors over product endorsement, which is particularly relevant when collaborating with pharmaceutical companies. By leveraging their credibility and reach, DOLs stand poised to catalyze transformative efforts in dengue prevention, heralding a new era of collective action and informed public health discourse.

## Appendix

Multimedia Appendix 1 Interview guide.

Multimedia Appendix 2 COREQ checklist.

Multimedia Appendix 3 Overview of the themes and corresponding quotes from semistructured interviews with digital opinion leaders.

## Abbreviations

COREQ: Consolidated Criteria for Reporting Qualitative Research
DOL: digital opinion leader
KOL: key opinion leader
NGO: nongovernmental organization
SMI: social media influencer
WHO: World Health Organization

## Data Availability

The datasets generated or analyzed during this study are available from the corresponding author on reasonable request.

## References

[1] Sharan A, Dasa V, Flickinger D, Bullock S, Martin J (2022). Moving from KOLs to DOLs - the changing influence of healthcare providers. J Orthop Exp Innov.

[2] Doggett J (2023). Are digital opinion leaders just influencers with medical qualifications?. CREATION.co.

[3] Huhn RH, Brantes Ferreira JB, Sabino de Freitas A, Leão FL (2018). The effects of social media opinion leaders’ recommendations on followers’ intention to buy. Revista Brasileira de Gestão de Negócios.

[4] Kaňková J, Binder A, Matthes J (2024). Helpful or harmful? Navigating the impact of social media influencers' health advice: insights from health expert content creators. BMC Public Health.

[5] Condran B, Gahagan J, Isfeld-Kiely H (2017). A scoping review of social media as a platform for multi-level sexual health promotion interventions. Can J Hum Sex.

[6] Mehmet M, Roberts R, Nayeem T (2020). Using digital and social media for health promotion: a social marketing approach for addressing co-morbid physical and mental health. Aust J Rural Health.

[7] Veale HJ, Sacks-Davis R, Weaver ER, Pedrana AE, Stoové MA, Hellard ME (2015). The use of social networking platforms for sexual health promotion: identifying key strategies for successful user engagement. BMC Public Health.

[8] Boon C, Golloub E (2021). How social media influencers helped the NYC public health system raise awareness of COVID-19 testing among historically disadvantaged populations. J Digital Soc Media Mark.

[9] Pöyry E, Reinikainen H, Luoma-Aho V (2022). The role of social media influencers in public health communication: case COVID-19 pandemic. Int J Strateg Commun.

[10] Klucarova S (2022). Do masks matter? Consumer perceptions of social media influencers who wear face masks amid the COVID-19 pandemic. Appl Psychol.

[11] Ortiz RR, Smith A, Coyne-Beasley T (2019). A systematic literature review to examine the potential for social media to impact HPV vaccine uptake and awareness, knowledge, and attitudes about HPV and HPV vaccination. Hum Vaccin Immunother.

[12] (2024). Dengue and severe dengue. World Health Organization.

[13] Kannan R, Soon LK, Govindasamy M (2019). Review on the role of social media for dengue prevention and monitoring. Appl Mech Mater.

[14] Du M, Jing W, Liu M, Liu J (2021). The global trends and regional differences in incidence of dengue infection from 1990 to 2019: an analysis from the global burden of disease study 2019. Infect Dis Ther.

[15] Lessa CL, Hodel KV, Gonçalves MS, Machado BA (2023). Dengue as a disease threatening global health: a narrative review focusing on Latin America and Brazil. Trop Med Infect Dis.

[16] Shepard DS, Coudeville L, Halasa YA, Zambrano B, Dayan GH (2011). Economic impact of dengue illness in the Americas. Am J Trop Med Hyg.

[17] Taylor L (2024). Dengue fever: Brazil rushes out vaccine as climate change fuels unprecedented surge. BMJ.

[18] Shepard DS, Undurraga EA, Halasa YA (2013). Economic and disease burden of dengue in Southeast Asia. PLoS Negl Trop Dis.

[19] Andraud M, Hens N, Marais C, Beutels P (2012). Dynamic epidemiological models for dengue transmission: a systematic review of structural approaches. PLoS One.

[20] Scott TW, Morrison AC (2010). Vector dynamics and transmission of dengue virus: implications for dengue surveillance and prevention strategies: vector dynamics and dengue prevention. Curr Top Microbiol Immunol.

[21] Achee NL, Gould F, Perkins TA, Reiner RC Jr, Morrison AC, Ritchie SA, Gubler DJ, Teyssou R, Scott TW (2015). A critical assessment of vector control for dengue prevention. PLoS Negl Trop Dis.

[22] Engaging communities to sustain dengue vector control. World Health Organization.

[23] Waickman AT, Newell K, Endy TP, Thomas SJ (2023). Biologics for dengue prevention: up-to-date. Expert Opin Biol Ther.

[24] Hadinegoro SR, Arredondo-García JL, Capeding MR, Deseda C, Chotpitayasunondh T, Dietze R, Hj Muhammad Ismail H, Reynales H, Limkittikul K, Rivera-Medina DM, Tran HN, Bouckenooghe A, Chansinghakul D, Cortés M, Fanouillere K, Forrat R, Frago C, Gailhardou S, Jackson N, Noriega F, Plennevaux E, Wartel TA, Zambrano B, Saville M (2015). Efficacy and long-term safety of a dengue vaccine in regions of endemic disease. N Engl J Med.

[25] Fatima K, Syed NI (2018). Dengvaxia controversy: impact on vaccine hesitancy. J Glob Health.

[26] Mendoza RU, Dayrit MM, Alfonso CR, Ong MM (2021). Public trust and the COVID-19 vaccination campaign: lessons from the Philippines as it emerges from the Dengvaxia controversy. Int J Health Plann Manage.

[27] Miras AP, Regencia ZJ, Baja ES (2023). 'I was terrified for my child': understanding the link between the Dengvaxia® controversy and the measles vaccine hesitancy in Pasay City, Philippines. J Public Health (Oxf).

[28] Wilder-Smith A (2024). TAK-003 dengue vaccine as a new tool to mitigate dengue in countries with a high disease burden. Lancet Global Health.

[29] Freedman DO (2023). A new dengue vaccine (TAK-003) now WHO recommended in endemic areas; what about travellers?. J Travel Med.

[30] (2024). WHO position paper on dengue vaccines – May 2024. World Health Organization.

[31] (2024). Dengue vaccine (TAK-003) GRADE tables for consideration by the Strategic Advisory Group of Experts (SAGE) on immunization. World Health Organization.

[32] Koduah A, Baatiema L, Kretchy IA, Agyepong IA, Danso-Appiah A, de Chavez AC, Ensor T, Mirzoev T (2022). Powers, engagements and resultant influences over the design and implementation of medicine pricing policies in Ghana. BMJ Glob Health.

[33] Kanwar A, Rahim M (2019). Social responsibilities of the global pharmaceuticals companies: towards an ethical health care paradigm. SSRN J.

[34] Braun V, Clarke V, Cooper H, Camic PM, Long DL, Panter AT, Rindskopf D, Sher KJ (2012). Thematic analysis. APA Handbook of Research Methods in Psychology, Vol. 2. Research Designs: Quantitative, Qualitative, Neuropsychological, and Biological.

[35] Finlay L (2021). Thematic analysis: the ‘good’, the ‘bad’ and the ‘ugly’. Eur J Qual Res Psychother.

[36] Tong A, Sainsbury P, Craig J (2007). Consolidated criteria for reporting qualitative research (COREQ): a 32-item checklist for interviews and focus groups. Int J Qual Health Care.

[37] Kaminski J (2024). Diffusion of innovation theory. Can J Nurs Inform.

[38] Katz E (1957). The two-step flow of communication: an up-to-date report on an hypothesis. Public Opinion Q.

[39] Khullar D (2022). Social media and medical misinformation: confronting new variants of an old problem. JAMA.

[40] Sadiq S (2024). UW study reveals how social media influencers profit from spreading misinformation. Oregon Public Broadcasting.

[41] Westberry C, Palmer XL, Potter L (2023). Social media and health misinformation: a literature review. Proceedings of the Future Technologies Conference 2023.

[42] Harff D, Bollen C, Schmuck D (2022). Responses to social media influencers’ misinformation about COVID-19: a pre-registered multiple-exposure experiment. Media Psychol.

[43] Rodrigues F, Ziade N, Jatuworapruk K, Caballero-Uribe CV, Khursheed T, Gupta L (2023). The impact of social media on vaccination: a narrative review. J Korean Med Sci.

[44] Sabbagh C, Boyland E, Hankey C, Parrett A (2020). Analysing credibility of UK social media influencers' weight-management blogs: a pilot study. Int J Environ Res Public Health.

[45] De Veirman M, Hudders L, Nelson MR (2019). What is influencer marketing and how does it target children? A review and direction for future research. Front Psychol.

[46] Vrontis D, Makrides A, Christofi M, Thrassou A (2021). Social media influencer marketing: a systematic review, integrative framework and future research agenda. Int J Consum Stud.

[47] Burt-D’Agnillo M (2022). Pro-metabolic brownies and anti-vaccine rhetoric: COVID-19 conspiracy theories, wellness influencers, and misinformation on Instagram. iJournal.

[48] Bonnevie E, Rosenberg SD, Kummeth C, Goldbarg J, Wartella E, Smyser J (2020). Using social media influencers to increase knowledge and positive attitudes toward the flu vaccine. PLoS One.

[49] Enke N, Borchers NS, Borchers NS (2021). Social media influencers in strategic communication: a conceptual framework for strategic social media influencer communication. Social Media Influencers in Strategic Communication.

[50] Guo M, Ganz O, Cruse B, Navarro M, Wagner D, Tate B, Delahanty J, Benoza G (2020). Keeping it fresh with hip-hop teens: promising targeting strategies for delivering public health messages to hard-to-reach audiences. Health Promot Pract.

[51] (2020). Systematic scoping review on social media monitoring methods and interventions relating to vaccine hesitancy. European Centre for Disease Prevention and Control.

[52] Nicholson MS, Leask J (2012). Lessons from an online debate about measles-mumps-rubella (MMR) immunization. Vaccine.

[53] Kostygina G, Tran H, Binns S, Szczypka G, Emery S, Vallone D, Hair E (2020). Boosting health campaign reach and engagement through use of social media influencers and memes. Social Media Soc.

[54] Lim MS, Molenaar A, Brennan L, Reid M, McCaffrey T (2022). Young adults' use of different social media platforms for health information: insights from web-based conversations. J Med Internet Res.

[55] Sumayyia MD, Al-Madaney MM, Almousawi FH (2019). Health information on social media. Perceptions, attitudes, and practices of patients and their companions. Saudi Med J.

[56] Neely S, Eldredge C, Sanders R (2021). Health information seeking behaviors on social media during the COVID-19 pandemic among American social networking site users: survey study. J Med Internet Res.

[57] Chou WY, Oh A, Klein WM (2018). Addressing health-related misinformation on social media. JAMA.

[58] Li Y, Wu S (2015). Dengue: what it is and why there is more. Sci Bull Sci Found Philipp.

[59] Wilder-Smith A (2020). Dengue vaccine development: status and future. Bundesgesundheitsblatt Gesundheitsforschung Gesundheitsschutz.

[60] Harapan H, Anwar S, Setiawan AM, Sasmono RT, Aceh Dengue Study (2016). Dengue vaccine acceptance and associated factors in Indonesia: a community-based cross-sectional survey in Aceh. Vaccine.

[61] (2024). Why do some vaccines work better than others?. American Society for Microbiology.

[62] Neale J (2016). Iterative categorization (IC): a systematic technique for analysing qualitative data. Addiction.

[63] Saunders B, Sim J, Kingstone T, Baker S, Waterfield J, Bartlam B, Burroughs H, Jinks C (2018). Saturation in qualitative research: exploring its conceptualization and operationalization. Qual Quant.

[64] Zahir A, Ullah A, Shah M, Mussawar A (2016). Community participation, dengue fever prevention and control practices in Swat, Pakistan. Int J MCH AIDS.

[65] Nguyen-Tien T, Probandari A, Ahmad RA (2019). Barriers to engaging communities in a dengue vector control program: an implementation research in an urban area in Hanoi City, Vietnam. Am J Trop Med Hyg.

