# Supplementary material for: The Role of Digital Opinion Leaders in Dengue Prevention Through Health Promotion and Public Health Collaboration: Qualitative Semistructured Interview Study
Source: J Med Internet Res. 2025 Apr 25;27:e70997. doi: 10.2196/70997 (PMC12064970; doi:10.2196/70997)
Supplement: Multimedia Appendix 1 [file jmir_v27i1e70997_app1.docx]

**Supplemental material A**

**Section 1: Introduction**

*Objective: Establish rapport with the respondent.*

1. Thank you for taking part in our interview. To start off, can you tell us about your
   1. Education level, medical/clinical/research credentials
   2. professional role, responsibilities, and primary place of practice?
2. What is your experience and involvement with dengue prevention and control?
   1. From a public health standpoint (integrated surveillance, outbreak preparedness, sustainable vector control, future vaccine implementation)
   2. From a research standpoint (operational and implementation research)
3. Can you describe your professional journey as an online content creator in the field of healthcare?
4. What were the triggers to start sharing content online?
5. What were your goals and objectives as an online creator?
6. What is your role today as a content creator and curator on social media platforms?
7. What has been your greatest achievement?
8. How did you establish your online presence and influence?
9. How do you manage your online presence alongside responsibilities in clinical practice?
   1. Have you heard of the term Digital Opinion Leader?
      1. Do you consider yourself as one? Why yes or why no

**Section 2: DOL journey and their followers**

*Objective: To gain insights into DOL’s operational methods, their sphere of influence and role in influencing practitioners/ followers*

**DOL’s operational methods**

1. When sharing healthcare-related content online…
   1. what is your primary intention?
   2. who is your target audience and what is your communication strategy for each segment?
      1. How do you share content online? Please give specific examples of your online presence (Probe all the channels, including talk show, podcasts, webinars, conferences…)
      2. What is your preferred format and why? (short videos, long videos, infographics, text posts, Instagram stories, images, Live streams)
      3. What is your preferred language? If using English versus local language, why?
      4. What is your preferred platform and why? (LinkedIn, FB, Instagram, TikTok, YouTube, Twitter, etc.).
   3. Can you share experiences of both successes and failures in your content sharing efforts?
   4. How do you stay engaged with your target audiences over time? How has your target audience grown?
2. How do you incorporate feedback from your audience into your content creation process?
   1. [Probe: Have there been instances where feedback lead to changes in your approach or messaging]
   2. What are your goals for expanding your audience in the future?
3. How often do you typically create and share healthcare related content related? How has it evolved since your very first online post? [Probe: frequency of posting, posting routine]
4. Given your medical background, how do you choose the healthcare-related content to share online? [Probe: are there specific areas within healthcare that you find resonate most with your audience?]
5. Can you provide examples of the type of healthcare content you typically share with your audience? Probe: new treatment, disease knowledge, misconceptions…
   1. What are you trying to address when you create these contents?
6. What are the content or approaches that tend to generate more engagements?
7. Is there any specific topic that you preferred?
   1. Why is this your preferred topic?
   2. Would you call yourself an advocate for a specific healthcare related topic? Which one and why?
8. How do you measure the impact and success of your content on your audience? Are there specific metrics or benchmarks you focus on to evaluate your influence and reach? [Probe: Follower count, engagement rate, impressions, reach, click through rate, conversion rate, content consistency, etc.]
   1. Can you share any examples of projects or campaigns where you measured and demonstrated your impact as an online influencer?
9. In your opinion, what are the essential skills and strategies that an online HCP influencer such as yourself should possess to craft engaging and influential content?

[Probe: story telling technique, content creation technique, audience engagement strategies, building credibility, platform utilisation, collaboration technique]

**DOL’s sphere of influence & Role in influencing practitioners and followers**

1. Can you provide insights into the demographics and characteristic of your followers or audience? [Probe: age group, professions, location, gender, and any difference between platform used]
2. How do you assess the level of trust and credibility your followers place in your opinions and recommendations?
3. What are the approaches you take to maintain this trust?
4. In your opinion, how has the role of HCPs evolved in the digital space and/ or social media platforms?
   1. What are the key changes or trends that you may have noticed lately in terms of HCPs involvement online?
   2. To what extent HCPs' influence or shape public's opinion on preventative care and vaccination on social media?
5. How do you see your role as a digital content creator in influencing your fellow practitioners or professionals in your fields?
6. Can you provide examples of instances where your digital presence has directly impacted or influenced other healthcare professionals?
7. What are your future goals and aspirations in terms of influencing other practitioners and shaping healthcare practices through your digital opinion leadership? [Probe: Future collaborations that aimed to influence best practices or advancements in healthcare]
8. And what about your goal towards the general public?

**Section 3: Dengue prevention content and vaccination**

*Objective: In the context of dengue prevention, understand their data and information sharing approach (e.g., how and what data are being disseminated), their source of information, their views/ role of vaccines, key information/ messages that would be critical/ relevant for dissemination with regards to dengue prevention​*

**Advocacy towards dengue prevention and vaccines**

1. In the field of preventative medicine, what has garnered most interest by your audience? Why?
   1. What is less of an interest? Why is it?
   2. Are there information that you would not share with your audience? Why?
      1. Are there specific words you would not employ? Which ones and Why?
   3. Probe: To what extent is dengue prevention interesting to your audience? Why is it interesting or not ?
      1. [If less interesting] What needs to be in place to increase the level of interest?
   4. Among your audience, which segment would be interested in dengue prevention? [Probe: How do you tailor your content to cater to different segment?]
2. To your best estimate, how significant is the dengue burden in your country/area of practice?
3. What is your overall perspective on dengue prevention?
   1. To what extent do you believe that dengue is a major public health concern? Why is that?
   2. What key information do you believe should be communicated to the public regarding dengue prevention?
4. As a healthcare professional posting content online, to what extent do you play a role in educating the public with prevention of infectious diseases such as dengue?
   1. And to what extent do you see HCPs online creators playing a role in the prevention and management of dengue fever? Kindly elaborate.
   2. As an expert, what is your role in educating other HCPs?
5. What are the current initiatives towards the prevention of dengue fever in the country that you feel are worth sharing with the public?
6. Are there specific emerging technologies or research methods that you find particularly promising in advancing dengue prevention efforts? Please elaborate
7. From your perspective, what role does dengue vaccines play in broader public health strategies for dengue prevention and control?
   1. Is this a topic you’d find relevant to share with your audience? Why is this relevant or less relevant to you?
8. Doctor, could you describe a time where you have shared content related to preventative measures?
   1. What type of content have you shared? [Probe: Statistics, prevention methods, vaccination…]
      1. How do your followers react or engage with content relating to infection diseases?
      2. How do these posts incite discussions, high engagement, shares?
      3. How important is interaction with your audience in shaping your messages?
   2. **[If not shared previously ask]** What is your opinion on sharing content on specific topics such as preventative care or vaccination?
      1. On a scale of 1 to 7, where 1 is unlikely and 7 is very likely, what is the likelihood for you to start sharing information on dengue prevention? Why do you say so?
      2. How would you approach the topic of infectious disease prevention such as dengue?
9. In your opinion, what is the ideal number of posts needed to drive a certain message? E.g., for the message of “dengue vaccine’s availability.”
   - 1. How frequently should you post to ensure audience awareness?
     2. How do you make sure their behaviour changes? Probe: Moving from awareness to getting oneself/family vaccinated
     3. What format would be most effective for your audience when sharing your content (E.g., long/short, pic/video, writing/picture, etc)
     4. Do you want to create the contents yourself or you're happy with reposts from trusted sources?
   1. Could you please describe the type of post on Dengue prevention you see as being reposted?
   2. Similarly, please also describe the type of post you have reposted or would be likelyto repost on Dengue prevention

1. What would be your approach in addressing concerns or misconceptions about dengue vaccination?
   1. Other non-HCP/KOLs (E.g., Non-doctor influencers) can also post about public health/prevention topics online. How does your role differ from them?

**Source of information**

1. In this rapid evolving field of healthcare, how do you stay informed about the latest research, treatments, and guidelines?
   1. What are the primary sources of information you rely on for staying updated?
   2. How do you ensure the accuracy and validity of the information you share? Could you describe your fact-checking process? [Probe: Researching new information online, discuss with other HCPs, drafting the posting, preparing the infographics]
2. Specifically for dengue prevention, what would be your step-by-step process to gather the information you would want to communicate online?
   1. What are all the channels (online and offline) that you would use to create your content around dengue prevention?
3. Have you ever collaborated with other HCPs or organisations to verify the accuracy of your content? Please describe.

**Section 4: Current or preferred practice of DOLs on collaboration**

*Objective: Explore current or preferred practice of DOLs on collaboration with the pharmaceutical industry and other stakeholders on dengue prevention and future vaccine education​.*

1. Could you provide an overview of how partnerships between digital content creators/influencers (DOL) and **government public health agencies** typically work, including types of content, activities and objectives involved in those collaborations?
   1. Do you see any specific areas for potential partnerships with **government public health agencies** to enhance dengue prevention initiatives and public education regarding future vaccines?
      1. How frequently would you collaborate with GOs?
      2. What factors influenced your decision to work with or avoid working with?
      3. How do you choose which partnerships align with your mission and values?
      4. In your experience, what are the key challenges that you have faced when collaborating with GOs?
2. Similarly, Could you provide an overview of how partnerships between digital content creators/influencers (DOL) and **NGOs** typically work, including types of content, activities and objectives involved in those collaborations?
   1. Do you see any specific areas for potential partnerships with **NGOs** to enhance dengue prevention initiatives and public education regarding future vaccines?
      1. How frequently would you collaborate with NGOs?
      2. What factors influenced your decision to work with or avoid working with?
      3. How do you choose which partnerships align with your mission and values?
      4. In your experience, what are the key challenges that you have faced when collaborating with NGOs?
3. And what about with pharmaceutical companies?
4. What are effective and ethical methods for pharma companies to collaborate with digital content creators/influencers in the context of Dengue prevention?
5. Can you share examples of pharmaceutical companies in which you have collaborated with or considered collaborating with? [Probe when was the collaboration?]
6. How frequently would you collaborate with pharmaceutical companies?
7. What factors influenced your decision to work with or avoid working with?
8. How do you choose which partnerships align with your mission and values?
9. In your experience, what are the key challenges that you have faced when collaborating with pharmaceutical companies?
10. What are your expectations in terms of remuneration model when collaborating with pharmaceutical companies?
11. Do you see any specific areas for potential partnerships with pharmaceutical companies to enhance dengue prevention initiatives and public education regarding future vaccines?
12. If so, can you elaborate on these potential partnerships?
13. Which pharmaceutical companies would you have in mind for collaboration on dengue prevention? Why?
14. Are there challenges or conflicts of interests that you consider when engaging in collaborations with other stakeholders in the health sector (such as government agencies, NGOS and the pharma industry) on dengue prevention and future vaccine education?
    1. **[If yes]** How would you navigate a potential conflict of interest to collaborate with a pharma company?
    2. What type of support and guidance would you require from pharmaceutical companies for you to successfully create an engaging content for your audience? [Probe: Future education, Prevention Efforts, Campaign Etc.]

**<<<<<<<<<<<<<<<<<<<END OF DISCUSSION GUIDE; THANK AND CLOSE>>>>>>>>>>>>>>>>>>>>>>>**
