# Supplementary material for: The Role of Digital Opinion Leaders in Dengue Prevention Through Health Promotion and Public Health Collaboration: Qualitative Semistructured Interview Study
Source: J Med Internet Res. 2025 Apr 25;27:e70997. doi: 10.2196/70997 (PMC12064970; doi:10.2196/70997)
Supplement: Multimedia Appendix 3 [file jmir_v27i1e70997_app3.docx]

**Multimedia Appendix 3.** Overview of the themes and corresponding quotes from semistructured interviews with digital opinion leaders (DOLs)

| Section, theme, and subthemes | | | Quotes |
| --- | --- | --- | --- |
| **Section 1: the increasing influence of DOLs in infectious disease prevention** | | | |
|  | **Influence of DOLs on infectious disease prevention** | | |
|  |  | Raising public awareness | “I see that building awareness is crucial. We can’t immediately push people to convert in a specific way. It often starts with creating awareness, even in non-medical aspects—simple, straightforward things to attract people and create the impression that.” [ID13, Southeast Asia]   - “The key is to educate them on what they do not know. Such information can be common knowledge for healthcare personnel, but not for the general public. The response is that they are more knowledgeable, but in terms of further action, nothing has been successful yet.”[ID17, Southeast Asia] |
|  |  | Dispelling misconceptions | - “For example, some people understand that once you are vaccinated you will not get sick, but this is incorrect. Actually, you can still get sick, but your symptoms would not be too severe. I want to get the fact out.” [ID17, Southeast Asia] |
|  |  | Serving as role models | - “I get the flu shot every year, and I’ve never posted about it. But if you ask me if it’s good to post as encouragement, sure.” [ID4, Latin America] - “I posted about my daughter getting the COVID-19 vaccine shot. It was a challenge, but in the end, many people thanked me and told me that from that moment on, they decided to vaccinate their children. I have done lots of things like this, and I don’t have any problem with that.” [ID7, Latin America] |
| **Section 2: current practices of DOLs contributing to dengue prevention** | | | |
|  | **Current challenges in dengue prevention and control** | | |
|  |  | Inadequate public awareness and knowledge of dengue and its preventive measures | - “None of my patients know about any medication to prevent dengue. If you’re talking about environmental aspects like Ridsect or similar products, nobody uses those anymore. Nobody uses the ‘kelambu’ [mosquito net]. I think the majority of our people are already relaxed; they are not worried about dengue and are not thinking much about prevention. Their households and neighbors are still contaminated with stagnant water.” [ID25, Southeast Asia] - “Traditional vector prevention measures, such as avoiding stagnant water and everything, are already well-known. I think that people have grown tired of this message, especially since we’ve been discussing this for 40 years without making any progress.” [ID8, Latin America] |
|  |  | Need for further governmental support | - “I raised concerns about the outbreak, but there was no clear response from the local health department.” [ID1, Southeast Asia] |
|  |  | Antivaccination sentiments, controversies, and the spread of false information | - “Our difficulty now is to get people to reduce the anti-vaccine movement because we come from a few years in which this gets much worse. It will be a battle to reinforce the population to trust vaccines again.”[ID5, Latin America] - “Oh, there are always fake news peddler. When I discuss anything about vaccines, there’s a lot of backlash. It’s like they are just waiting to be triggered.” [ID28, Southeast Asia] |
|  | **Topics to discuss for dengue prevention and sources of information** | | |
|  |  | Promoting vector control strategies and raising awareness of dengue symptoms | - “For instance, regarding dengue fever, we promote a healthy lifestyle, following government guidelines such as the 3M approach—covering water containers, draining water from baths, and cleaning mosquito breeding grounds. We follow these practices extensively.” [ID2, Southeast Asia] - “We need to have better campaigns for the population to talk about how to control the vector, which is very difficult.” [ID6, Latin America] - “No, they’re not really interested in dengue prevention. I think, in general, people already have good and sufficient knowledge about dengue. It’s been talked about repeatedly, even in schools. So when I make content about it, it’s like, ‘Hmm, we already know that.’ Although some people still watch, the interest isn’t very strong.” [ID29, Southeast Asia] |
|  |  | The pivotal role of vaccines in mitigating the dengue burden | - “The majority of people are not against the vaccines, but they show vaccine hesitancy. They listen to a lot of information, and they don’t know exactly what to believe.” [ID4, Latin America] - “We can create content debunking myths on vaccines…We need to change their mindsets, so we explain why their belief is wrong.” [ID32, Southeast Asia] |
|  |  | Application of new technologies in dengue prevention | - “The latest technologies are Wolbachia and vaccination. These two should be promoted more.” [ID1, Southeast Asia] |
|  |  | Heavy reliance on scientific journals and authoritative websites as information sources | - “When creating content, it’s definitely journals. Because journals are valid, verified, and adhering to international standards. So, they are suitable as a source of information.” [ID3, Southeast Asia] - “There are probably three main sources that have information related to this. First is, of course, the Ministry of Health. The second is the [national] Paediatric Society. The third is the [national] Doctors Association. These are the three medical professional bodies that issue guidelines related to dengue vaccination and management, both in children and adults.” [ID3, Southeast Asia] |
| **Section 3. exploring ways of collaboration to strengthen dengue prevention** | | | |
|  | **Types of collaboration** | | |
|  |  | Collaboration with pharmaceutical companies on health education and product awareness | - “But I’ve had previous partnerships with pharmaceutical companies, but they were nonexclusive. It still depends on what will be discussed, but I’m open to collaboration.” [ID32, Southeast Asia] - “I collaborate with [name of a pharmaceutical company], discussing [name of a product]. Then with [name of another pharmaceutical company], talking about environmental cleanliness. There were a few more products.” [ID1, Southeast Asia] |
|  |  | Collaboration with NGOs^a^ on advancing nutrition and hygiene practice | - “I collaborated with [name of an international NGO] on issues related to stunting, nutrition, and promoting handwashing during the COVID-19 pandemic.” [ID3, Southeast Asia] |
|  |  | Collaboration with government and health agencies on public health education initiatives | - “With the Ministry of Health, I’ve collaborated with them several times. This includes programs related to mosquitoes and Wolbachia [for dengue prevention], as well as various vaccination campaigns, including the one for HPV.” [ID3, Southeast Asia] - “Perhaps producing content, talking about, and answering questions about vaccines—who has been vaccinated, who has not been vaccinated, and who cannot be vaccinated.” [ID9, Latin America] |
|  | **Factors considered for collaboration** | | |
|  |  | Adherence to ethical standards | - “So, I need to double-check what they’re selling and ensure that what they want me to say aligns with best medical practices and the value of the product.” [ID27, Southeast Asia] - “I need to make sure that I am entering something ethical because we know there is an ethical board and a code that we cannot breach.” [ID9, Latin America] - “It must adhere to ethical standards.” [ID3, Southeast Asia] |
|  |  | Alignment with DOLs’ values | - “If the campaign is focused on educating people about the disease rather than selling the product, [it’s fine]. If it’s too product-centred, I would say no. We make sure the campaign is all about education.” [ID29, Southeast Asia] - “[It depends on] if we have the same goal or not. Also, if my role would be benefiting for the course or not, if not, then I will not work with them.” [ID18, Southeast Asia] |
|  |  | Credibility of scientific evidence supporting the initiatives | - “My personal approach is to always rely on scientific evidence, and that’s what I’m doing.” [ID11, Latin America] - “I believe that the product needs to be aligned with scientific evidence. The product must have been vetted by ANVISA, FDA, EMA, and other regulatory agencies, and the project must align with the codes of medical ethics and medical advertising.” [ID7, Latin America] |
|  |  | Preferences on compensation in the collaboration | - “We have a price list. I have a pricing table for Stories and a pricing table for posts...there’s a whole pricing guide.” [ID8, Latin America] - “Yes. Usually, speaking engagements for doctors come with an honorarium. There’s usually a specific designated amount offered by the company.” [ID33, Southeast Asia] |
